# Microbial sequencing to improve individual and population health

**DOI:** 10.1186/s13073-014-0103-5

**Published:** 2014-11-19

**Authors:** Sharon J Peacock, George M Weinstock

**Affiliations:** Department of Medicine, University of Cambridge, Box 157 Addenbrooke’s Hospital, Hills Road, Cambridge, CB2 0QQ UK; The Wellcome Trust Sanger Institute, Wellcome Trust Genome Campus, Hinxton, Cambridge CB10 1SA UK; The Jackson Laboratory for Genomic Medicine, At the University of Connecticut Health Center, Administrative Services Building, 263 Farmington Ave, Farmington, CT 06030 USA

## Abstract

Recent advances in sequencing technologies are changing the face of infectious disease investigation and control. Personalized anti-infective therapies and surveillance of emergent pathogen outbreaks are just two examples of the potential benefits of merging the fields of genomics and infectious diseases.

## Editorial

This *Genome Biology* and *Genome Medicine* collaborative special issue on the genomics of infectious diseases is very timely. Vaccination, access to clean water, and antimicrobial drugs have all changed the relationship between humans and pathogens, resulting in a marked increase in life expectancy. Yet, infectious diseases continue to take their toll on human health worldwide, and events such as the recent Ebola outbreak in West Africa serve as a sharp reminder of how fragile any success is in the control of pathogens. A more insidious but pervasive threat to human health is the emergence and dissemination of antimicrobial resistance among numerous pathogens, paralleled by a decline in antimicrobial drug discovery. Advances in sequencing technologies have resulted in the availability of instruments that can be operated in a clinical environment, together with high-throughput platforms that can be used to define pathogens at the population level. These technologies have numerous potential applications for the control of infectious diseases.

Sequencing will bring improvements in the detection and control of outbreaks associated with multidrug-resistant and other pathogens in hospitals and the community [1]. Confirmation of an outbreak could lead to earlier implementation of interventions that bring the outbreak to a close [2]. Conversely, excluding an outbreak with confidence will reduce unnecessary infection control interventions [3]. Pathogen sequencing will be used to tailor individual patient prescribing. Capillary sequencing of the human immunodeficiency virus (HIV) is already used to guide the treatment of patients who are HIV positive, but newer sequencing technologies will bring the added benefit of detecting resistant variants present as a minority of the HIV population in a given individual. In tuberculosis (TB), sequencing technologies will be used to predict antimicrobial resistance of the causative agent, *Mycobacterium tuberculosis* [4]. This will bring the greatest benefit to patients with multidrug-resistant and extensively drug-resistant TB (against which first- and second-line drugs are not effective), because conventional testing of second-line drugs is lengthy. Accurate prescribing could lead to more rapid resolution of infection and reduced risk of onward transmission. Genome sequencing also defines transmission of *M. tuberculosis* between individuals with greater resolution and certainty than was previously possible [5].

Passive surveillance using sequence data generated for clinical use would provide an overview of the emergence and spread of antimicrobial resistance. Active genomic surveillance of key human pathogens would provide an early warning system for outbreaks, inform vaccine strategies through tracking of vaccine escape, and detect the emergence of new clones that harbor known or novel virulence determinants. Sequencing is being used to identify reservoirs of antimicrobial-resistance genes in hospitals, other healthcare facilities, the community, and livestock farming, as well as common transmission pathways between them. Finding pinch-points to stop transmission between reservoirs could limit the dissemination of antimicrobial resistance. Sequencing also provides insights into the emergence of infectious diseases. For example, reconstruction of the early dynamics of the HIV pandemic using sequence data and statistical approaches identified Kinshasa in the 1920s as the focus of early transmission and the source of pre-1960 pandemic viruses elsewhere [6]. Sequencing of the more recently emerged Middle East respiratory syndrome coronavirus and comparison of sequence data for isolates from humans and dromedary camels has been cited as evidence for the role of camels as a reservoir [7].

Sequencing also has a role in drug discovery pathways, the laboratory evaluation of lead compounds, and the clinical phases of drug evaluation. For example, in 2005, in the first published use of 454 pyrosequencing, the F0 subunit of ATP synthase was identified as the target of bedaquiline [8]. Bedaquiline subsequently became the first representative of the only novel class of anti-TB agents to be approved in 40 years. Sequencing of *M. tuberculosis* during clinical trials can be used to distinguish exogenous re-infection from a relapse of the primary infection, which is crucial to assess the efficacy of study drugs. Sequencing technologies will also underpin clinical trials evaluating the effect of a therapeutic alteration of the microbiome in a range of conditions. The benefit derived from duodenal infusion of donor feces in patients with recurrent *Clostridium difficile* infection provides proof-of-principle for clinical utility [9]. Extending this to other diseases will need to be supported by detailed genomic analyses of the human microbiota, together with a better understanding of the interactions between the native or medically altered microbiome and host immunity.

Several challenges remain before microbial sequencing becomes routine for diagnostic and public health microbiology laboratories. A suite of software tools will be required to convert sequence data into a format that is relevant and useful for clinicians and infection control teams. New methods to handle and process ever-expanding pathogen-specific microbial genome databases will also be needed, including global and region-specific listings of gene mutations associated with drug resistance. It is also essential that existing mechanisms for the development of standard operating procedures and accreditation of laboratory methods be applied to microbial sequencing. Working within a tightly controlled diagnostic laboratory will reduce errors (for example, through sample tracking) and allow data to be handled within an existing framework that protects patient confidentiality.

Further technological advances are also required to reduce the turnaround time between taking a clinical sample and generating sequence data. Refinements such as extracting DNA directly from a bacterial colony on a culture plate can reduce the processing time by up to a day [10]. However, the need to culture the sample to obtain a pure growth of bacteria from which to purify DNA prior to sequencing, rather than performing direct sequencing on the sample, means that timelines are still tied to bacteriology methods that were developed more than a hundred years ago. Regardless, the enthusiasm for direct sequencing of clinical samples should be tempered by the probable reality of doing so. Most samples sent to a diagnostic laboratory are currently reported out as ‘no growth’ or, through the use of selective culture media that target specific pathogens, ‘no significant growth’. In a brave new world where all samples are sequenced as the primary method for pathogen detection, it may prove the case that the majority of samples will be sequence-positive. Re-defining what data can be disregarded and what might represent new and important findings will take at least a generation of microbiologists to resolve.

## Abbreviations

HIV: Human immunodeficiency virus
TB: Tuberculosis
